# TRIM22 activates NF-κB signaling in glioblastoma by accelerating the degradation of IκBα

**DOI:** 10.1038/s41418-020-00606-w

**Published:** 2020-08-19

**Authors:** Jianxiong Ji, Kaikai Ding, Tao Luo, Xin Zhang, Anjing Chen, Di Zhang, Gang Li, Frits Thorsen, Bin Huang, Xingang Li, Jian Wang

**Affiliations:** 1 Department of Neurosurgery, Qilu Hospital and Institute of Brain and Brain-Inspired Science, Cheeloo College of Medicine, Shandong University, Jinan, China; 2 Shandong Key Laboratory of Brain Function Remodeling, Jinan, China; 3 Department of Biomedicine, University of Bergen, Jonas Lies vei 91, 5009 Bergen, Norway; 4 Molecular Imaging Center, University of Bergen, Jonas Lies vei 91, 5009 Bergen, Norway

**Keywords:** CNS cancer, Oncogenes, Ubiquitin ligases

## Abstract

NF-κB signaling plays a critical role in tumor growth and treatment resistance in GBM as in many other cancers. However, the molecular mechanisms underlying high, constitutive NF-κB activity in GBM remains to be elucidated. Here, we screened a panel of tripartite motif (TRIM) family proteins and identified TRIM22 as a potential activator of NF-κB using an NF-κB driven luciferase reporter construct in GBM cell lines. Knockout of *TRIM22* using Cas9-sgRNAs led to reduced GBM cell proliferation, while *TRIM22* overexpression enhanced proliferation of cell populations, in vitro and in an orthotopic xenograft model. However, two TRIM22 mutants, one with a critical RING-finger domain deletion and the other with amino acid changes at two active sites of RING E3 ligase (C15/18A), were both unable to promote GBM cell proliferation over controls, thus implicating E3 ligase activity in the growth-promoting properties of TRIM22. Co-immunoprecipitations demonstrated that TRIM22 bound a negative regulator of NF-κB, NF-κB inhibitor alpha (IκBα), and accelerated its degradation by inducing K48-linked ubiquitination. TRIM22 also formed a complex with the NF-κB upstream regulator IKKγ and promoted K63-linked ubiquitination, which led to the phosphorylation of both IKKα/β and IκBα. Expression of a non-phosphorylation mutant, srIκBα, inhibited the growth-promoting properties of TRIM22 in GBM cell lines. Finally, TRIM22 was increased in a cohort of primary GBM samples on a tissue microarray, and high expression of TRIM22 correlated with other clinical parameters associated with progressive gliomas, such as wild-type IDH1 status. In summary, our study revealed that TRIM22 activated NF-κB signaling through posttranslational modification of two critical regulators of NF-κB signaling in GBM cells.

## Introduction

The NF-κB pathway, originally characterized within the context of the immune system, has been implicated in many hallmarks of cancer development, including cellular proliferation, angiogenesis, and resistance to therapy [1–3]. As in other malignancies, high, constitutive NF-κB activity has been observed in human glioblastoma (GBM). It has been found to promote mesenchymal differentiation and therapy resistance in the disease [4], and also plays a central role in many other active oncogenic pathways in GBM [5–10]. Expression profiling and molecular analysis has demonstrated that GBMs with mesenchymal features exhibit elevated levels of NF-κB pathway genes, such as *RELB* and *TRADD* [11]. However, mutation or amplification of NF-κB signaling subunits is rare in tumors, suggesting that aberrant activation of NF-κB signaling in GBM may be attributed to deregulation of the pathway or oncogenes.

Ubiquitination, one of the most common and important types of posttranslational modification, has been implicated in many diseases including cancers [12, 13]. The most well-studied polyubiquitin chain types include lysine 48 (K48) and lysine 63 (K63) linkages. K48-linked polyubiquitin chains predominantly target proteins for degradation, while K63 chains regulate kinase activity, signal transduction, and endocytosis [14, 15]. Over the past decade, posttranslational modification, especially ubiquitin (Ub) modification, has emerged as a crucial participant in NF-κB activation [16]. Following stimulation, signaling intermediaries, such as TNF receptor-associated factors and receptor interacting protein, are rapidly modified with K63-linked poly-Ub chains, leading to activation of downstream kinases, such as TGF-β-activated kinase 1 and IKK complexes that participate in NF-κB signaling. The IKK complex is made up of two catalytic subunits (IKKα and IKKβ) and a noncatalytic regulatory subunit (IKKγ) [16–20]. Transcriptional activation of NF-κB occurs when the IKK complex becomes activated and phosphorylates IκB proteins. Phosphorylation of IκBα frees NF-κB, which is translocated to the nucleus, while phosphorylated IκBα is targeted for degradation after modification with K48-linked Ub chains [21].

One subfamily of the RING type E3 Ub ligases, the tripartite motif (TRIM) proteins, is emerging as a key regulator in the development of diverse cancers by modulating transcriptional activity of NF-κB, including GBM [13]. TRIM40 was reported to physically binds to NEDD8 and promotes the neddylation of IKKγ to inhibit NF-κB activation in gastrointestinal carcinomas [22]. TRIM24 has also been shown to significantly alter transcriptional activity of NF-κB in EGFRvIII-driven GBM cells [23]. Here, using NF-κB response reporters and public databases, we further investigated the role of TRIM proteins in the development of GBM. We screened a panel of TRIM proteins using an NF-κB driven luciferase reporter construct and identified TRIM22 as an activator of NF-κB signaling in GBM cells. TRIM22, rarely investigated in most human cancers, regulates biological processes in various cell types through NF-κB signaling, including macrophages [24, 25], neurons [26], and human embryonic kidney 293 T cells (HEK293T) [27, 28]. We found it not only promoted K48-linked ubiquitination of IκBα by directly associating with it, but also enhanced K63-linked ubiquitination of IKKγ. Thus, TRIM22 both activates the IKK complex and promotes degradation of IκBα, thereby leading to high transcriptional activity of NF-κB in GBM cells.

## Materials and methods

### Ethics statement

All primary glioma tissue samples (*n* = 112) and matching clinical data were obtained from the Department of Neurosurgery at Qilu Hospital of Shandong University [29]. Nonneoplastic brain tissue samples (NBT; *n* = 10) were obtained from the Department of Pathology at Qilu Hospital of Shandong University. All experiments and the use of human tissues were approved by the Research Ethics Committee of Shandong University and the Ethics Committee of Qilu Hospital in accordance with the Declaration of Helsinki (for humans) and the U.S. Public Health Service Policy on Human Care and Use of Laboratory Animals (2015 reprint; for mice). Written informed consent was obtained from all adult patients.

### Cell culture

Human GBM cell lines (U87MG, U118MG and LN229) and the human embryonic kidney cell line 293 (HEK293) were purchased from the American Type Culture Collection. All human cell lines were authenticated and submitted for short tandem repeat analysis (Cell Cook Biotech Co. LTD; Guangzhou, China). Primary GBM#P3 cells and BG5 glioma stem cells (GSC) were kind gifts provided by Prof. Rolf Bjerkvig (University of Bergen, Norway). Cells (U87MG, U118MG, LN229, and HEK293) were maintained in the Dulbecco’s modified Eagle’s medium (DMEM; Life Technologies/Thermo Fisher Scientific; Waltham, MA, USA) supplemented with 10% fetal bovine serum (Thermo Fisher Scientific). Cells (GBM#P3 and GSC BG5) were cultured in serum-free DMEM/F12 medium (Gibco/Thermo Fisher Scientific) supplemented with 2% B27 Neuro Mix (Thermo Fisher Scientific), epidermal growth factor (20 ng/mL; Thermo Fisher Scientific), and basic fibroblast growth factor (10 ng/mL; Thermo Fisher Scientific). Cells were maintained at 37 °C in a humidified chamber containing 5% CO_2_.

### Transient transfection, lentiviral infection, and Cas9-sgRNA knockout

Transient transfections for siRNAs and plasmids were performed with Lipofectamine 2000 or 3000 (Thermo Fisher Scientific) as we previously described [30]. The sequences of siRNAs used are listed in Supplementary Table S1, and plasmids used are listed in Supplementary Table S2.

The Puro-3×Flag-hCas9 and single-guide RNA (sgRNAs) lentiviruses were designed and constructed by OBiO Technology Company (Shanghai, China). Lentivirus containing Cas9 and sgRNAs (sg-scramble, sg-*TRIM22*-1 or sg-*TRIM22*-2) were introduced into U87MG, LN229, GBM#P3, and BG5 cells. The sequences of sgRNAs used are listed in Supplementary Table S1.

In addition, lentiviral constructs for ectopic expression of full-length *TRIM22* (Flag-TRIM22-FL; OBiO Technology), a RING-finger domain deletion mutant of *TRIM22* (Flag-TRIM22-ΔRING; OBiO Technology), a C15/18A mutant of *TRIM22* (Flag-TRIM22-C15/18A; OBiO Technology), and an S32/S36A mutant of *IκBα* (srIκBα; OBiO Technology) were also used to infect cells.

After 48 h, infected cells were cultured in media containing puromycin (2 μg/mL; Thermo Fisher Scientific) for 2 weeks to select for stable expression.

### Subcellular fractionation

Nuclear and cytoplasmic fractions from LN229 and U118MG were isolated using Nuclear and Cytoplasmic Extraction Reagents (Thermo Fisher Scientific), according to the manufacturer’s instructions. Subcellular distribution of proteins was determined using western blot analysis. GAPDH and Histone H3 served as loading controls for cytosolic and nuclear fractions, respectively.

### Real-time quantitative RT-PCR (qRT-PCR)

Total RNA was extracted from cells using TRIzol Reagent (Thermo Fisher Scientific). RNA (2 µg) was reverse transcribed into cDNA using the ReverTra Ace qPCR RT Kit (Toyobo Life Science; Shanghai, China) according to the manufacturer’s protocols. Quantitative PCR was performed using the SYBR premix Ex Taq (Takara; Tokyo, Japan) on the Real-Time PCR Detection System (480II, Roche; Basel, Switzerland). GAPDH served as the internal control. Primers used for PCR are listed in Supplementary Table S3.

### Immunohistochemistry (IHC), immunofluorescence (IF), and western blotting (WB)

IHC, IF, and WB were performed as previously described [29]. The scoring system for TRIM22 IHC staining and all antibodies used are described in Supplementary Materials and Methods.

### Co-immunoprecipitation (Co-IP)

Co-IPs were performed as previously described [29]. Briefly, cells were lysed in IP lysis buffer (Thermo Fisher Scientific) containing a protease inhibitor cocktail (1%, Sigma-Aldrich; St Louis, MO, USA). Total lysates (200 µg; 1 µg/µL) were incubated with primary antibodies (4 µL) or IgG (4 µL) overnight at 4 °C with gentle shaking followed by Protein A/G magnetic beads (Thermo Fisher Scientific) for 2 h at room temperature. The immunoprecipitated complexes were immunoblotted. The antibodies used are listed in Supplementary Materials and Methods.

### Cycloheximide (CHX) chase

LN229 and U87MG cells were infected with lentivirus containing Cas9 and sgRNAs targeting *TRIM22* (OBiO Technology). LN229 and U118MG cells were infected with lentivirus for ectopic expression of Flag-TRIM22-FL, Flag-TRIM22-C15/18A, or Flag-TRIM22-ΔRING (OBiO Technology). After selection, CHX (25 μg/mL; Apexbio; Houston, TX, USA) was introduced to the culture medium to inhibit translation, and cell lysates were prepared at the indicated times. Protein (20 μg) was examined using western blot analysis.

### Cell number counting

Cells with target gene knockout or ectopic overexpression (1 × 10^5^/well) were seeded into six-well plates. Cells were collected through trypsinization and counted every 24 h. Cells were counted in three wells to obtain an average count, and each experiment was performed in three independent biological replicates.

### Luciferase reporter assays

The NF-κB firefly-luciferase and renilla reporter constructs (100 ng each, Promega; Madison, WI, USA) were co-transfected into modified U87MG, U118MG, and LN229 cells using Lipofectamine 3000 (Thermo Fisher Scientific). After 24 h, luciferase activities were examined using the Dual-Luciferase Reporter Assay Kit (Promega). Renilla activity was used to normalize luciferase reporter activity. The promoterless firefly-luciferase vector pGL4.15 served as the negative control (NC). Assays were performed on cells in three wells for each experiment to obtain an average count, and in three independent biological replicates.

### In vivo and in vitro ubiquitination assay

To assess in vivo ubiquitination, modified cells were treated with 20 μM MG132 (Apexbio; Houston, TX, USA) for 6 h before lysis, followed by co-IP and western blot analysis. For in vitro ubiquitination assays, HA-IκBα proteins purified from HEK293 cells were incubated with UBE1 (100 ng), UbcH5a (150 ng), human recombinant Ub (5 μg, Boston Biochem; Cambridge, MA, USA) in the absence or presence of Flag-TRIM22-FL or Flag-TRIM22-ΔRING purified proteins with ubiquitination reaction buffer (Boston Biochem) at 30 °C for 90 min. Co-IPs were performed on the incubation mixture using an anti-HA antibody followed by western blot analysis using an anti-K48-linkage specific polyubiquitin antibody.

### Animal studies

Athymic nude mice (male, 4-week old; GemPharmatech Co., Ltd; Nanjing, China) were randomly divided into five animals per group. Luciferase-expressing human glioma cell lines (3 × 10^5^ cells suspended in 10 μL PBS) were implanted into the frontal lobes of nude mice using a stereotactic apparatus (KDS310, KD Scientific; Holliston, MA, USA). Tumor growth was examined at 6, 12, and 24 days after implantation using bioluminescence imaging (IVIS spectrum in vivo imaging system, PerkinElmer; Hopkinton, MA, USA). Animals were euthanized by cervical dislocation when they displayed any symptoms of continuous discomfort, such as severe hunchback posture, decreased activity, apathy, dragging legs, or more than 20% weight loss. Mouse brains were harvested and examined through hematoxylin and eosin and IHC/IF staining.

### Database (oncomine)

Oncomine (https://www.oncomine.org/) was used to compare mRNA expression levels between GBM and nonneoplastic tissue samples for *TRIM5*, *TRIM21*, *TRIM22*, and *TRIM38* in TCGA Brain datasets and *TRIM56* in the Sun Brain dataset.

### Statistical analysis

All experiments were performed in at least three independent biological replicates and reported as the mean ± the standard error of the mean. The statistical significance was calculated utilizing an unpaired two-tailed Student’s *t* test for direct comparisons and ANOVA for multigroup comparisons. Survival curves were estimated using the Kaplan–Meier method and compared using the log-rank test. Correlation between *TRIM22* expression levels and clinicopathological factors was determined using the two-tailed *χ*^2^ test or the Fisher’s exact test. Statistical analysis was conducted using GraphPad Prism version 7.00 software for Windows (GraphPad; La Jolla, CA, USA). Differences were considered as statistically significant when *P* values were < 0.05.

## Results

### Identification of TRIM22 as a positive regulator of NF-κB signaling

**Fig. 1:**
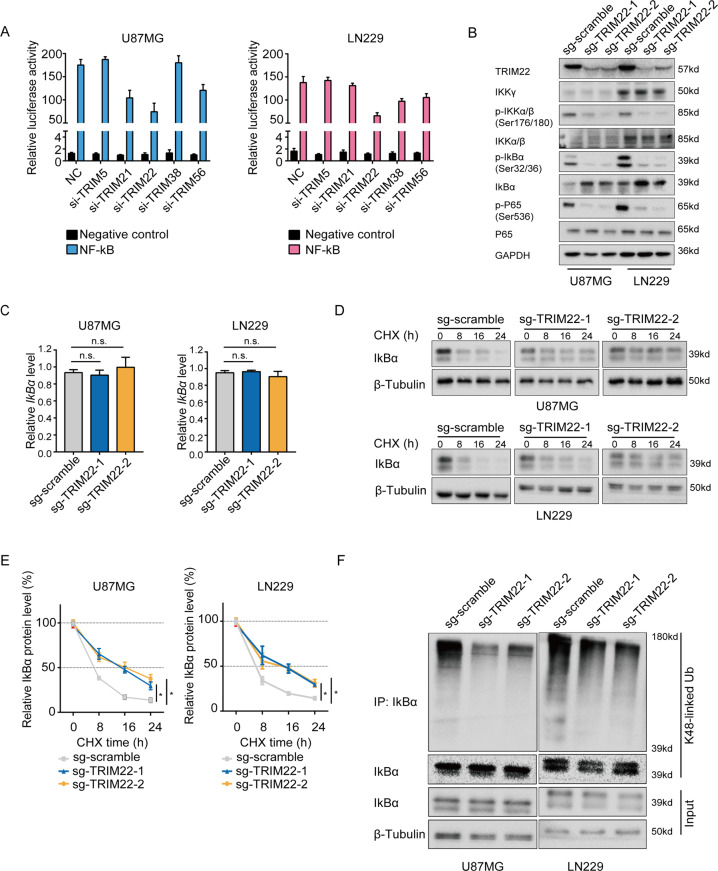
TRIM22 actives NF-κB signaling via K48-linked ubiquitination of IκBα. **a** Luciferase activity from U87MG and LN229 cells transfected with siRNAs of *TRIM5*, *TRIM21*, *TRIM22*, *TRIM38,* or *TRIM56*, along with a reporter plasmid carrying the NF-κB promoter relative to negative control. **b** Western blot analysis to evaluate core kinases in the NF-κB pathway in lysates (20 µg) prepared from *TRIM22* knockout cells. GAPDH was used as the loading control. **c** qRT-PCR analysis of *IκBα* mRNA levels in modified U87MG and LN229 cells relative to the control sg-scramble. GAPDH was used to normalize samples. **d** Western blot analysis of IκBα protein in TRIM22-depleted cells treated with cycloheximide (CHX; 25 μg/mL) for 0, 8, 16, and 24 h. **e** Decay curve of IκBα levels normalized to β-tubulin and to 0 h at the indicated time points from CHX experiments. **f** Western blot analysis of IPs performed with antibody to IκBα to detect endogenous IκBα ubiquitination from indicated cells. Antibody for K48-linked polyubiquitin was used on the western blot. Student’s *t* test: n.s. not significant, *P < 0.05.

To identify novel NF-κB signaling modulators, we screened the activity of a panel of TRIM proteins using an NF-κB-dependent transcriptional reporter containing five copies of an NF-κB response element (NRE) located upstream of luciferase. We chose to analyze five TRIM proteins, TRIM5, TRIM21, TRIM22, TRIM38, and TRIM56, in our luciferase assay based on two criteria. First, the expression levels of the genes, based on molecular data in public datasets in Oncomine, were significantly elevated in GBM relative to NBT samples (Supplementary Fig. S1A). Second, these five *TRIM* genes have not yet been reported to have a role in the development of human gliomas. We transfected siRNAs targeting each of these genes with the luciferase reporter construct into U87MG and LN229 cells and measured luciferase activity. *TRIM22* siRNA decreased reporter activity the most among the five *TRIM* genes in both U87MG and LN229 cells compared with the NC group (Fig. 1a). Using CRISPR/Cas9 technology, we knocked out *TRIM22* in both cell lines and found luciferase activity to be significantly reduced (Supplementary Fig. S2A).

To elucidate the mechanism underlying reduced NF-κB activity in *TRIM22* knockout cells, we used western blot analysis to examine total protein and phosphorylated levels of core kinases involved in canonical NF-κB signaling. Phosphorylated levels of the proteins that examined IKKα/β (Ser176/180), IκBα (Ser32/36), and P65 (Ser536) were uniformly decreased in *TRIM22* knockout cell lines. However, total protein levels remained unchanged, except in the case of IκBα, where they increased in response to *TRIM22* knockout (Fig. 1b and Supplementary Fig. S3A).

To understand how loss of TRIM22 might contribute to increased levels of IκBα protein, we first examined *IκBα* mRNA levels. *IκBα* mRNA levels did not change significantly with *TRIM22* knockout in U87MG and LN229 cells (Fig. 1c). This result indicated that IκBα protein might be stabilized in *TRIM22* knockout cells. Therefore, we determined the half-life of IκBα by treating modified U87MG and LN229 cells with CHX. The half-life of IκBα was prolonged by ~8 h in U87MG-sg-*TRIM22* and LN229-sg-*TRIM22* cells compared to the sg-scramble groups (Fig. 1d, e). Finally, K48-linked ubiquitination of IκBα in control and modified U87MG and LN229 cells paralleled TRIM22 expression; endogenous K48-linked ubiquitination of IκBα was decreased with knockout of *TRIM22* (Fig. 1f). Taken together, these data suggested that deletion of *TRIM22* enhanced IκBα protein stability through loss of proteasomal-mediated protein degradation.

### *TRIM22* deletion inhibits GBM cell proliferation

**Fig. 2:**
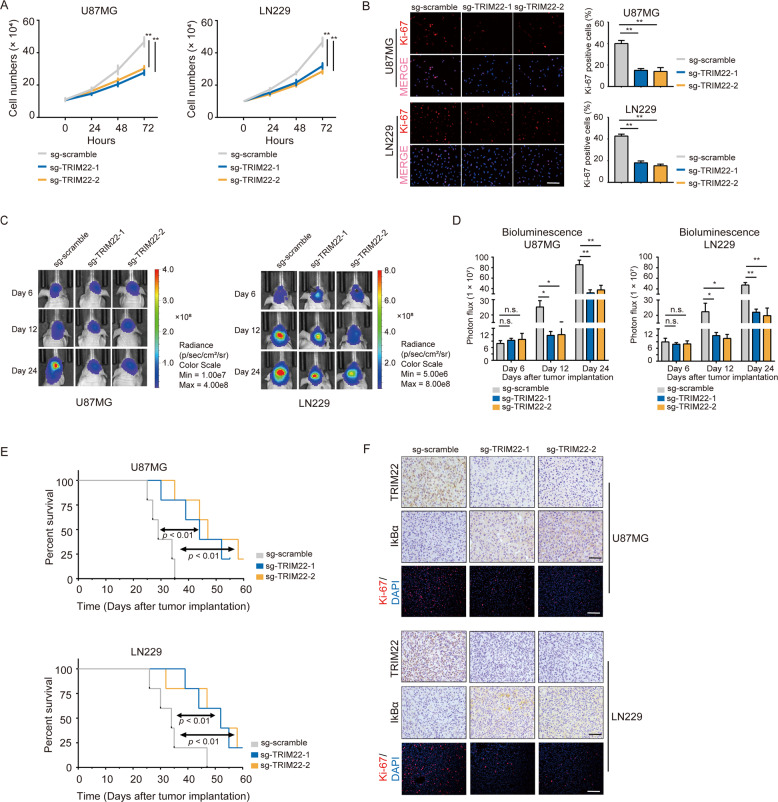
Knockout of *TRIM22* inhibits proliferation of GBM in vitro and in vivo. **a** Growth curves generated using cell counting over 72 h for the cells indicated. **b** Representative images and quantification of Ki-67 immunofluorescence staining from modified U87MG and LN229 cells. **c**, **d** Images and quantification of in vivo bioluminescence imaging of U87MG- and LN229-NC and -sg-*TRIM22*-1 and -2 derived xenografts at the indicated time points. **e** Kaplan–Meier survival analysis performed with survival data from indicated groups. Log-rank test, *P* < 0.01. **f** Representative images of IHC staining for TRIM22 and IκBα, and IF staining for Ki-67 levels in xenograft sections from NC and sg-*TRIM22*-1 and -2 groups. Scale bars, 50 μm for IHC, and 100 μm for Ki-67 IF staining. Student’s *t* test: n.s. not significant, *P < 0.05, **P < 0.01.

NF-κB signaling is considered to be a pivotal factor in inducing genes that promote cell survival and proliferation [5]. Our previous work also demonstrated a growth-promoting role for NF-κB in GBMs [29]. To test whether TRIM22 is involved in regulating GBM cell proliferation, growth curves were generated for *TRIM22* knockout U87MG and LN229 cells and compared to controls in vitro. Growth curves generated over 72 h revealed that *TRIM22* knockout significantly attenuated proliferation of U87MG and LN229 cells relative to control cell lines (Fig. 2a). Reduced Ki-67 IF staining in knockdown cells further demonstrated that TRIM22 promoted GBM cell proliferation in vitro (~50%; Fig. 2b).

We also examined tumor growth in vivo. Luciferase-expressing modified and control cell lines were orthotopically implanted in mice, and tumor growth was monitored/quantified using bioluminescence. Tumors derived from U87MG- and LN229-sg-*TRIM22*-1 or -2 cells were slower growing than the sg-scramble control counterparts (Fig. 2c, d). Survival time of U87MG- and LN229-sg-*TRIM22*-1 or -2 tumor-bearing animals was also prolonged relative to controls (U87MG: 44 or 47 days vs. 29 days, sg-*TRIM22*-1 or -2 vs. sg-scramble, respectively, *P* < 0.01; LN229: 52 or 52 days vs. 34 days, sg-*TRIM22*-1 or -2 vs. sg-scramble, respectively, *P* < 0.01; Fig. 2e). IHC staining performed on sections from xenografts showed that IκBα expression levels increased in the absence of TRIM22, while Ki-67-IF staining was decreased (Fig. 2f and Supplementary Fig. S4A, B). Collectively, these data indicated that TRIM22 promoted growth of GBM tumors in vivo.

### E3 ligase activity is required for TRIM22-mediated GBM cell proliferation

**Fig. 3:**
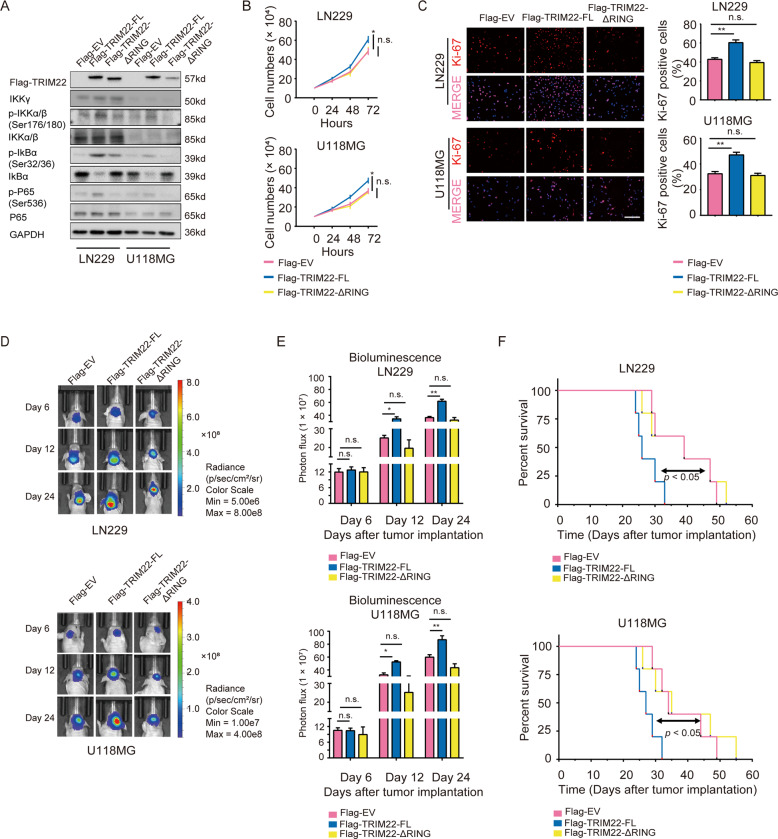
Overexpression of TRIM22 promotes proliferation of GBM in vitro and in vivo via its RING domain. **a** Western blot analysis to evaluate components of the NF-κB pathway in lysates prepared from LN229- and U118MG-EV, TRIM22-FL and TRIM22-ΔRING cell populations. GAPDH was used as the loading control. **b** Growth curves generated using cell counting and **c** Ki-67 immunofluorescence staining for transfected cells in **a**. Scale bars, 100 μm. **d**, **e** Representative images and quantification of in vivo luciferase bioluminescence from indicated cells orthotopically implanted into the brains of nude mice at day 6, 12, and 24 after injection. **f** Kaplan–Meier analysis of survival for tumor-bearing mice implanted with indicated cells. Log-rank test, *P* < 0.05. Student’s *t* test: n.s. not significant, *P < 0.05, **P < 0.01.

TRIM22 is a RING domain E3 Ub ligase mediating protein ubiquitination [31]. We therefore constructed two E3 ligase defective TRIM22 mutants, one with a RING domain deletion (TRIM22-ΔRING) and the other with the amino acid changes at conserved cysteines C15 and C18 to alanines (TRIM22-C15/18A) [32–34], to determine whether the growth-promoting activity of TRIM22 is associated with its E3 Ub ligase activity. Flag-tagged TRIM22-ΔRING, TRIM22-C15/18A, *TRIM22*-full length (TRIM22-FL), and empty vector (EV) were transfected into LN229 and U118MG. Phosphorylated IKKα/β (Ser176/180), IκBα (Ser32/36) and P65 (Ser536) were elevated, while total IκBα was decreased, in cells overexpressing TRIM22 (TRIM22-FL) compared with EV groups (Fig. 3a and Supplementary Fig. S5A). However, each of these proteins remained unchanged in TRIM22-ΔRING/-C15/18A groups compared with EV groups (Fig. 3a and Supplementary Fig. S5A). TRIM22-FL also significantly increased cell proliferation (Fig. 3b, c and Supplementary Fig. S5B). In contrast, the RING domain deletion and C15/18A mutants did not enhance cell proliferation over controls (Fig. 3b, c and Supplementary Fig. S5B).

These data were confirmed in orthotopic xenograft models (Fig. 3d, e and Supplementary Fig. S5C, D). Survival time of tumor-bearing mice was shorter in LN229- and U118MG-TRIM22-FL groups (LN229: 26 vs. 39 days, TRIM22-FL vs. EV, respectively, *P* < 0.05; U118MG: 27 vs. 34 days, TRIM22-FL vs. EV, respectively, *P* < 0.05; Fig. 3f), but remained stable in LN229- and U118MG-TRIM22-ΔRING (LN229: 39 vs. 39 days, TRIM22-ΔRING vs. EV, respectively, *P* = n.s.; U118MG: 35 vs. 34 days, TRIM22-ΔRING vs. EV, respectively, *P* = n.s.; Fig. 3f) relative to EV groups. Taken together, these data suggested that the TRIM22 plays an important role in driving tumor growth possibly through its E3 Ub ligase activity.

### TRIM22 ubiquitinates IκBα

**Fig. 4:**
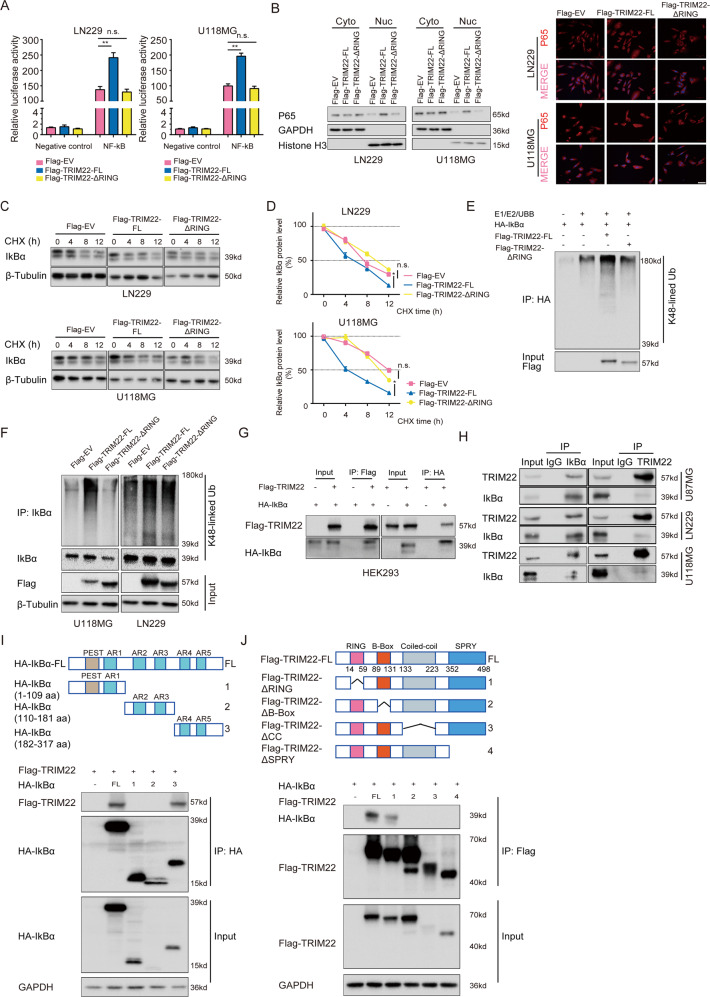
TRIM22 ubiquitinates IκBα. **a** Luciferase activity for NF-κB luciferase or control reporter constructs in modified LN229 and U118MG cells. **b** Western blot analysis of cytoplasmic (Cyto) and nuclear (Nuc) fractions prepared from indicated cells. Immunofluorescence for P65 in modified LN229 and U118MG cells showing cellular localization. Scale bars, 20 μm. **c** Western blot to detect IκBα levels after 0, 4, 8, and 12 h of cycloheximide (CHX; 25 μg/mL) treatment in modified LN229 and U118MG cells compared with controls. **d** Line graph showing IκBα protein levels normalized to β-tubulin and to 0 h at the indicated time points. **e** Western blot of IP of IκBα incubated with anti-K48-linkage specific polyubiquitin antibody to detect ubiquitination of IκBα in an in vitro assay. **f** In vivo ubiquitination assay of IκBα. **g** Western blot analysis of co-IPs performed on lysates prepared from HEK293 cells transfected with Flag-TRIM22 and HA-IκBα. **h** Western blot analysis of co-IPs performed using anti-IκBα or -TRIM22 antibody on lysates prepared from U87MG, LN229, and U118MG cells. **i** Schematic representation of wild-type IκBα and the indicated deletion mutants. Western blot analysis of co-IPs performed on lysates prepared from HEK293 cells transfected with Flag-TRIM22 alone or together with indicated HA-IκBα constructs. Upper panels represent co-IPs performed with anti-HA; lower panels represent input protein. **j** Schematic representation of wild-type TRIM22 and the indicated deletion mutants. Western blot analysis of co-IPs performed on lysates prepared from HEK293 cells transfected with HA-IκBα alone or together with indicated Flag-TRIM22 constructs. Student’s *t* test: n.s. not significant, *P < 0.05, **P < 0.01.

To explore whether the E3 ligase activity is critical for TRIM22-mediated activation of NF-κB signaling, we tested TRIM22-ΔRING and TRIM22-C15/18A in NF-κB luciferase reporter assays compared with TRIM22-FL and EV groups. NF-κB signaling activity increased significantly in LN229 and U118MG cells transfected with TRIM22-FL, but remained unchanged in cells expressing TRIM22-ΔRING/-C15/18A (Fig. 4a and Supplementary Fig. S5E). Elevated nuclear accumulation of P65 in LN229- and U118MG-TRIM22-FL groups was the further demonstration of an enhanced activation of NF-κB signaling, but remained unchanged in cells expressing TRIM22-ΔRING/-C15/18A (Fig. 4b and Supplementary Fig. S5F). In CHX studies, the half-life of IκBα was decreased by ~4 h in LN229-TRIM22-FL cells and ~8 h in LN229-TRIM22-FL cells compared to EV groups, while changes in LN229- and U118MG-TRIM22-ΔRING/-C15/18A were not significant (Fig. 4c, d and Supplementary Fig. S5G, H). Furthermore, both in vitro and in vivo, TRIM22-FL overexpression induced endogenous K48-linked ubiquitination of IκBα, while the RING domain deletion and C15/18A mutants had no effect (Fig. 4e, f and Supplementary Fig. S5I).

To test whether TRIM22 directly binds to IκBα, we performed co-IPs using lysates prepared from HEK293 transiently transfected with Flag-tagged TRIM22 and HA-tagged IκBα expression vectors. Immunoprecipitation with anti-FLAG or anti-HA antibodies brought down both Flag-tagged TRIM22 and HA-tagged IκBα indicating that the two tagged proteins were associated with each other in HEK293 cells (Fig. 4g). An endogenous physical interaction between the two proteins was confirmed in co-IPs using U87MG, LN229, and U118MG cell lysates (Fig. 4h). To investigate the functional domains responsible for their interaction, a series of deletion mutant constructs for TRIM22 and IκBα were expressed in cells, and co-IPs were performed. AR4 and AR5 domains in IκBα, or amino acids 182–317, brought down TRIM22. In TRIM22, amino acids 89–131 (B-Box domain), 133–223 (coiled-coil domain), and 352–498 (SPRY domain) were necessary to bring down IκBα (Fig. 4i, j). Interestingly, although the TRIM22 ring domain mutant was less efficient at binding IκBα, the domain was not essential for the two proteins to interact. Thus, TRIM22 might promote the development of human glioma by facilitating proteasomal-mediated degradation of IκBα, a negative regulator of NF-κB signaling.

### TRIM22 promotes K63-linked ubiquitination of IKKγ

**Fig. 5:**
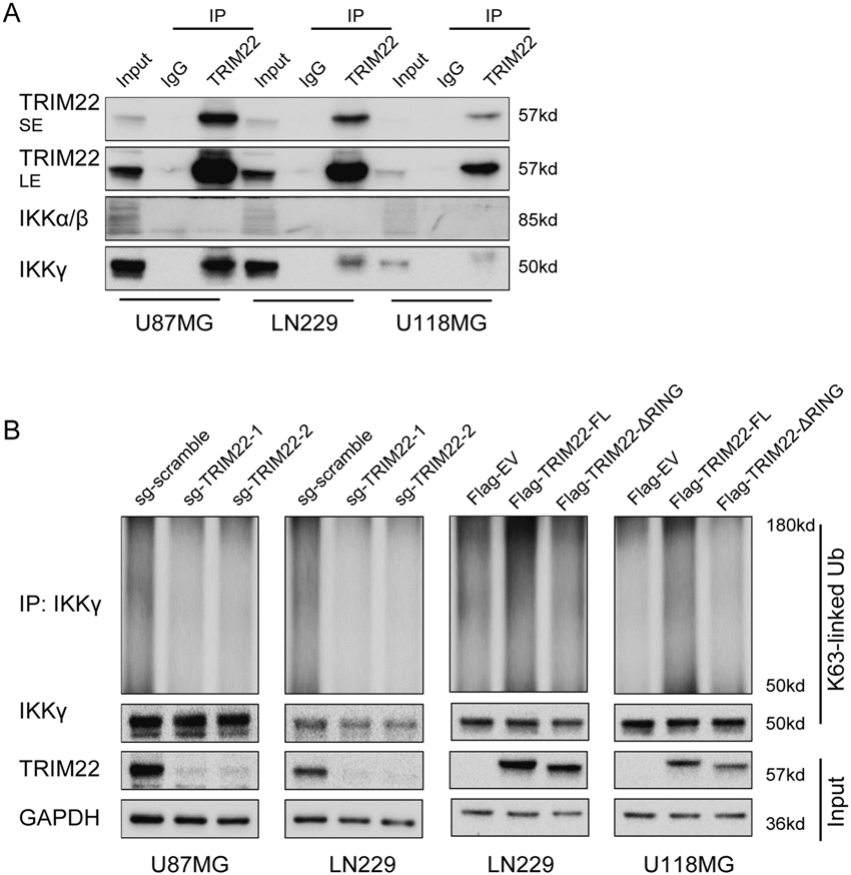
TRIM22 promotes K63-linked ubiquitination of IKKγ. **a** Western blot analysis of co-IPs to demonstrate association of TRIM22 with IKKα/β or IKKγ in U87MG, LN229, and U118MG cells. **b** Western blot analysis for IKKγ of co-IPs with K63-linkage specific polyubiquitin antibody in the indicated modified U87MG, LN229, and U118MG cells.

Loss of TRIM22 also led to decreased phosphorylation of IKKα/β (Ser176/180), IκBα (Ser32/36), and P65, indicating that it may have some role in the phosphorylation and thus activation of these proteins, which are mediators of so-called canonical NF-κB signaling (Fig. 1b). We therefore performed co-IP assays with anti-TRIM22 antibodies to determine whether any of these proteins were associated with TRIM22 in GBM cell lines. We found that IKKγ, rather than IKKα/β, was associated with TRIM22 in GBM cells (Fig. 5a). We furthermore found that K63 ubiquitination levels of IKKγ paralleled TRIM22 protein levels (Fig. 5b). In summary, these data suggested that TRIM22 might regulate NF-κB activation through activation of the IKK complex.

### IκBα mediates the tumor growth-promoting effects of TRIM22

**Fig. 6:**
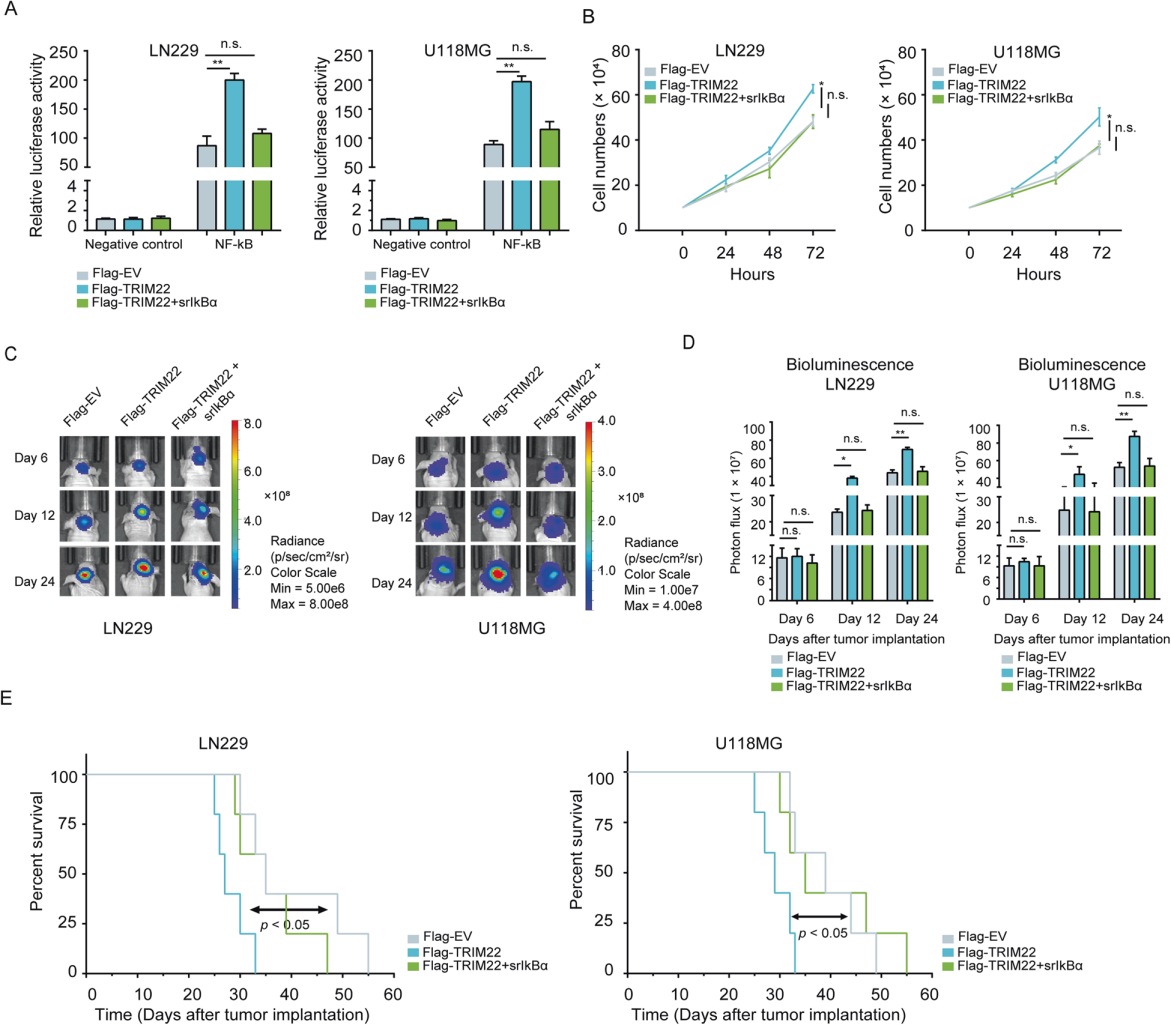
TRIM22 promotes the growth of GBM via IκBα. **a** Luciferase activity in modified LN229 and U118MG cells transfected with NF-κB luciferase reporter constructs. **b** Growth curves generated with cell counting performed over 72 h. **c**, **d** In vivo bioluminescence imaging and quantification of modified LN229 and U118MG cells derived xenografts at the indicated time points. **e** Kaplan–Meier survival analysis performed with survival data of tumor-bearing animals implanted with the indicated cells. Log-rank test, *P* = n.s., *P* < 0.05. Student’s *t* test: n.s. not significant, *P < 0.05, **P < 0.01.

To determine whether TRIM22 activates NF-κB signaling through IκBα, we introduced a construct expressing an IκBα phosphorylation mutant (S32/36A; srIκBα) into LN229 and U118MG cells. The substitution of S32 and S36 with alanine leads to a constitutively unphosphorylated state in srIκBα, which prevents its degradation and subsequent NF-κB activation [35]. Thus, we first tested whether TRIM22 induced NF-κB activity in the presence of srIκBα in glioma cells, using the NF-κB luciferase reporter construct. In LN229- and U118MG-TRIM22-FL cells, NF-κB transcriptional activity was increased relative to controls (~2×; Fig. 6a). However, NF-κB activity did not increase in TRIM22 overexpressing cells in the presence of srIκBα (Fig. 6a).

In cell number counting assays, srIκBα blocked the increase in cell proliferation mediated by overexpression of TRIM22 in LN229 and U118MG cells relative to controls (Fig. 6b). The growth-promoting properties of TRIM22 were also shown to be mediated by IκBα in an orthotopic tumor model. LN229- and U118MG-TRIM22-FL tumors were greater in size compared with EV groups, but this effect was abolished by srIκBα overexpression (Fig. 6c, d). Overall survival was also correspondingly reduced in mice bearing LN229- and U118MG-TRIM22-FL tumors relative to control mice (LN229: 27 vs. 35 days, TRIM22-FL vs. EV, respectively, *P* < 0.05; U118MG: 29 vs. 39 days, TRIM22-FL vs EV, respectively, *P* < 0.05; Fig. 6e). However, srIκBα overexpression in the context of TRIM22 overexpression brought overall survival back to nearly control group levels (LN229: 35 vs. 39 days, TRIM22-FL + srIκBα vs. EV, respectively, *P* = n.s.; U118MG: 35 vs. 35 days, TRIM22-FL + srIκBα vs. EV, respectively, *P* = n.s.; Fig. 6e). These results indicated that IκBα is a key effector in TRIM22-promoted growth of GBM cell populations in vitro and in vivo.

### TRIM22 predicts higher grade of human glioma malignancy

**Fig. 7:**
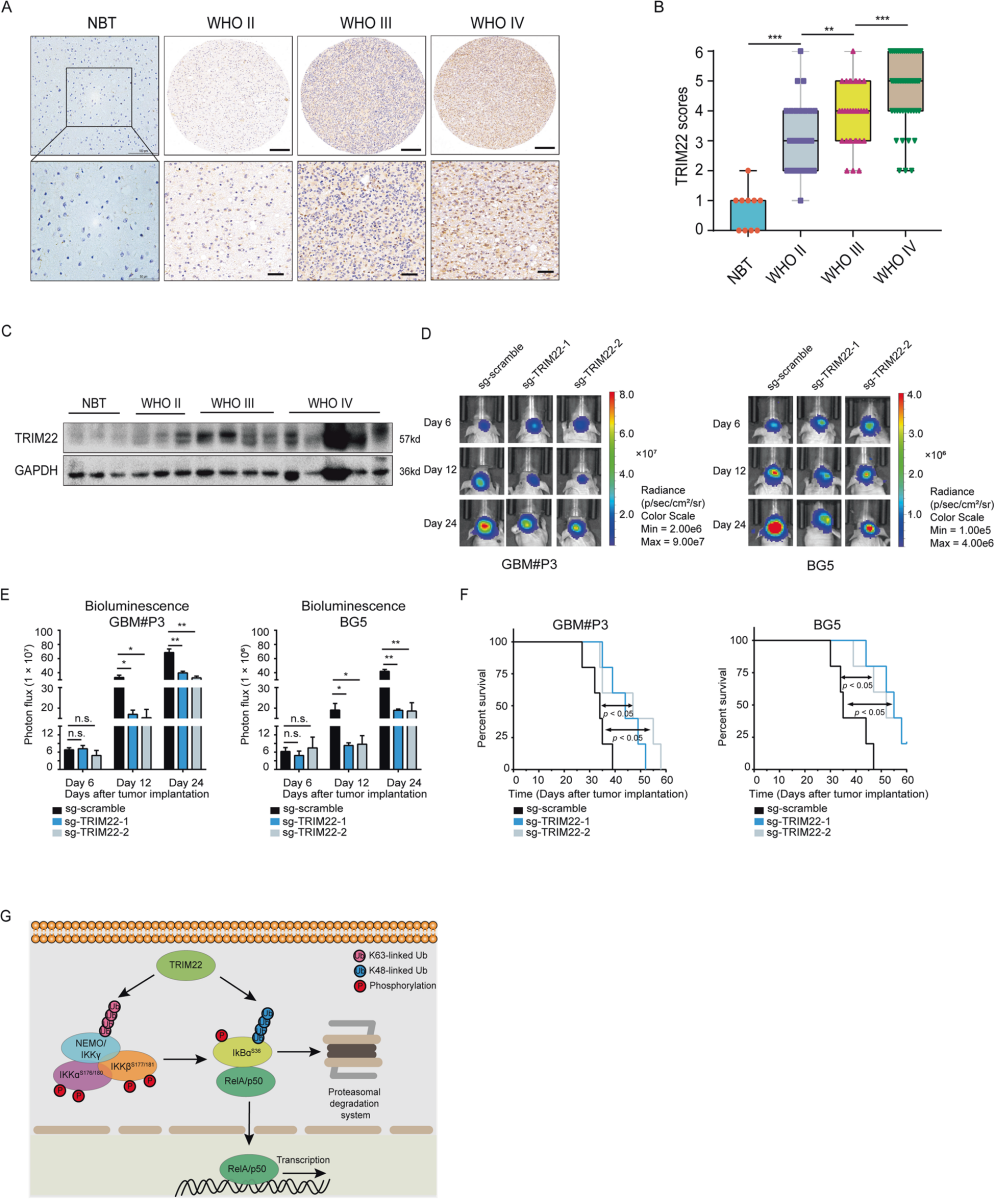
*TRIM22* is highly expressed in GBM samples and correlates with other clinical parameters associated with higher grade glioma. **a** Images of TRIM22 IHC performed on WHO grade II–IV glioma tissue microarrays and nonneoplastic brain tissues (NBT). Representative image of NBT was from the frontal lobe. Scale bar = 100 μm (top) or 50 μm (bottom). For glioma tissue microarrays, scale bar = 200 μm (top) or 50 μm (bottom). **b** Graphic representation of scoring performed on IHC staining for TRIM22 in primary samples from glioma tissue microarrays and NBTs. **c** Western blot analysis of TRIM22 protein levels in primary glioma tissues (*n* = 12) and nonneoplastic brain tissue samples (*n* = 3). **d**, **e** In vivo bioluminescence imaging and quantification of modified GBM#P3 and GSC#BG5 cells derived xenografts at the indicated time points. **f** Kaplan–Meier survival analysis performed with survival data from indicated cells. Log-rank test, *P* < 0.05. **g** A graphical model for TRIM22-mediated NF-κB activation and GBM growth, showing interaction of TRIM22 with critical components of the pathway, including IκBα which normally sequesters the transcription activating complex in the cytoplasm. TRIM22 targets IκBα for proteasomal-mediated degradation and activates the IKK complex through K63-linked ubiquitination of IKKγ/NEMO, which leads to further disruption of IκBα from the complex. Student’s *t* test: n.s. not significant, *P < 0.05, **P < 0.01, ***P < 0.001.

To characterize its role in the progression of human gliomas, we evaluated TRIM22 protein levels in a cohort of primary gliomas on a tissue microarray and NBT samples using IHC. The histology and distribution of the samples was as follows: WHO grade II (*n* = 30), WHO grade III (*n* = 31), WHO grade IV (*n* = 51, GBMs), and nonneoplastic brain (NBT, *n* = 10). TRIM22 was preferentially expressed in high grade gliomas (*n* = 82; HGG, WHO grade III-IV) compared with low grade gliomas (*n* = 30; LGG, WHO grade II; *P* < 0.001; Fig. 7a, b and Supplementary Table S4). Expression in NBT samples was nearly absent (Fig. 7a, b and Supplementary Fig. S6A). WB of lysates prepared from primary tumors (*n* = 12; WHO grades II–IV) and NBT samples (*n* = 3) confirmed the overexpression of TRIM22 in human gliomas (Fig. 7c). Finally, increased expression of TRIM22 correlated with other features of more aggressive gliomas, including wild-type IDH1 (*P* < 0.001) and wild-type ATRX (*P* = 0.0054).

We also performed experiments with GBM#P3 and GSC#BG5 cells, which more faithfully retain the genetic features of the matching primary GBM [36]. Knockout of *TRIM22* in GBM#P3 cells led to decreased tumor growth in vivo (Fig. 7d, e) and prolonged the survival time of tumor-bearing mice (GBM#P3: 44 or 47 days vs. 34 days, sg-*TRIM22*-1 or -2 vs. sg-scramble, respectively, *P* < 0.05; BG5: 55 or 52 days vs. 35 days, sg-*TRIM22*-1 or -2 vs. sg-scramble, respectively, *P* < 0.05; Fig. 7f). These data are in agreement with a growth-promoting role for TRIM22 in the development of human gliomas.

## Discussion

Targeting NF-κB signaling is emerging as a promising therapeutic strategy for GBM. To date, studies regarding NF-κB as a potential therapeutic target have focused on nonspecific compounds or inhibitors of IKK, which impact many of the pathways central to the malignant phenotype of GBM [37]. However, NF-κB inhibitors initiate a broad range of responses and side effects. Thus, glioma patients may benefit from inhibition of specific factors upstream of various mediators of NF-κB signaling [38]. In this study, we identified TRIM22 as a potential modulator of NF-κB signaling, by screening activity from an NF-κB-dependent transcriptional luciferase reporter construct in response to knockout of five TRIM genes overexpressed in GBM. We demonstrated that TRIM22 promotes glioma cell proliferation in vitro and tumor formation in vivo, and that these properties are linked to its intrinsic E3 Ub ligase activity. TRIM22 aids in the generation of K48-linked Ub conjugates of IκBα, a key negative regulator of NF-κB signaling, and thus promotes enhanced proteasomal-mediated degradation of the protein. TRIM22 also associates and generates K63-linked Ub conjugates of IKKγ, which in turn activate IKK complexes and subsequently degrade IκBα (model in Fig. 7g). TRIM22 exhibited increased expression in primary human GBM specimens, and high TRIM22 correlated with factors associated with higher grade gliomas. Our study therefore revealed a novel regulatory mechanism for IκBα degradation and NF-κB activation, and a critical role for TRIM22 in glioma tumorigenesis.

TRIM22 was first identified as an IFN-induced protein and also found to be a transcriptional target gene of TP53 [39, 40]. Although the proximal promoter of *TRIM22* is not p53-responsive, a functional enhancer-like element was found in intron 1 of the *TRIM22* gene. TP53, which is frequently deregulated in GBM, plays a central role in the classification of GBM molecular subtypes and GBM progression [41]. We found that TRIM22 is preferentially expressed in U87MG and A172 cell lines (P53 wild type) compared with LN229 and U118MG cell lines (P53 mutated; data not shown here). The significance of the association between TRIM22 and *P53* status in human glioma requires further investigation.

TRIM22 contains a conserved RING domain, which suggests that it has a potential role as an E3 Ub ligase involved in posttranscriptional modification of certain proteins [31, 32, 42]. Specificity of the Ub conjugation system is derived from the direct association of the E3 ligase with its substrates. Here, we demonstrated that TRIM22 interacted with both IκBα and IKKγ, causing ubiquitination which was E3 ligase activity-dependent. Overexpression of TRIM22, which promoted glioma cell proliferation in vivo and in vitro, was blocked through deletion of the RING domain or substitution of two conserved cysteine sites by alanine. Thus, we determined that the E3 ligase activity of TRIM22 was required for its function. However, it has been reported that the C terminal SPRY domain [27, 32, 43], which is in a region that we found to be necessary for the interaction between TRIM22 and IκBα, is also important for TRIM22-mediated biological functions. Therefore, further study is necessary to determine which region of TRIM22 might be relevant to the development of human gliomas.

IKKγ, also known as the NF-κB essential modulator, is a regulatory subunit of the IκB kinase complex and has a critical role in the activation of NF-κB [44]. IκBα binds strongly to NF-κB and sequesters it in the cytoplasm in resting cells. Upon stimulation, the IκB kinase/IKK complex phosphorylates IκBα at serine 32/36. NF-κB becomes active as phosphorylated IκBα is released, and undergoes K48-linked polyubiquitination and proteasome-dependent degradation [37]. Previous studies have demonstrated that the F-box protein β-TrCP1 is involved in the proteasomal-mediated degradation of IκBα [45]. However, a subsequent study showed that IκBα was still degraded when β-TrCP1 was knocked out, suggesting that other E3 ligases might target IκBα for proteasomal-mediated degradation [45]. Therefore, we screened the activity of a panel of TRIM proteins using the NF-κB response reporter, which led to the identification of TRIM22 as a potential regulator of NF-κB signaling. We demonstrated that TRIM22-targeted IκBα for proteasomal-mediated degradation and further enhanced this protein degradation by modifying K63-linked polyubiquitin chains on IKKγ, despite the existence of linear and K27-linked ubiquitination of IKKγ [46, 47].

In summary, our study revealed a novel regulatory mechanism of NF-κB activation, in which TRIM22, an E3 ligase, promotes K48- and K63-linked ubiquitination of IκBα and IKKγ, respectively. TRIM22 induced NF-κB signaling in GBM, which drives tumor growth and progression. Finally, our study defines TRIM22 as a candidate therapeutic target. Pharmaceutical inhibition of its E3 ligase activity or the interaction between these three proteins may provide a promising strategy for the treatment of GBM.

## Supplementary information

Supplementary Figure S1

Supplementary Figure S2

Supplementary Figure S3

Supplementary Figure S4

Supplementary Figure S5

Supplementary Figure S6

Supplementary Table S1-S4

Supplementary Materials and Methods, Figure Legends
