# Supplementary figures and images for: TRIM22 activates NF-κB signaling in glioblastoma by accelerating the degradation of IκBα
Source: Cell Death Differ. 2020 Aug 19;28(1):367–81. doi: 10.1038/s41418-020-00606-w (PMC7853150; doi:10.1038/s41418-020-00606-w)

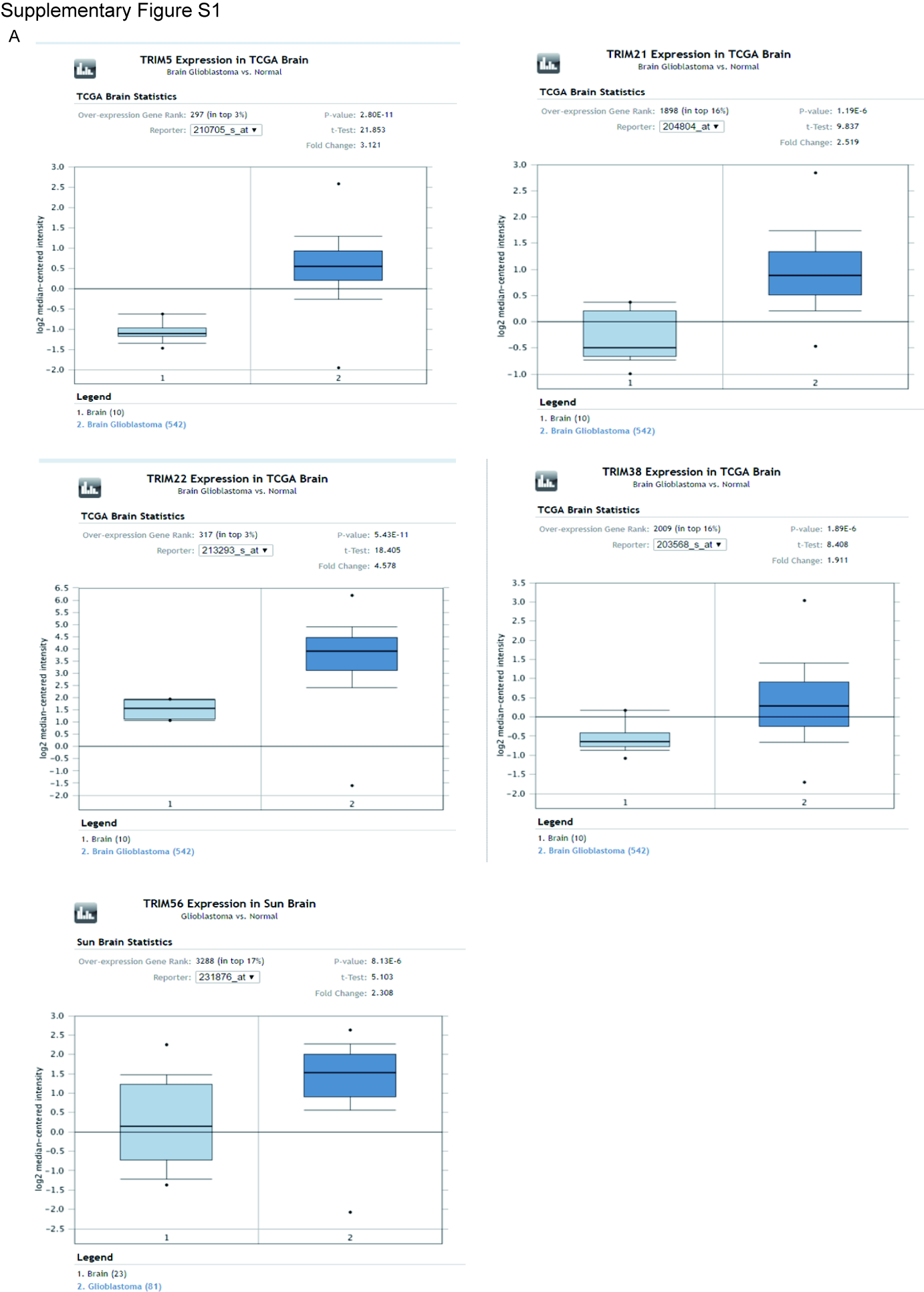

Supplement: Supplementary file 1 — Supplementary Figure S1 [file 41418_2020_606_MOESM1_ESM.tif]

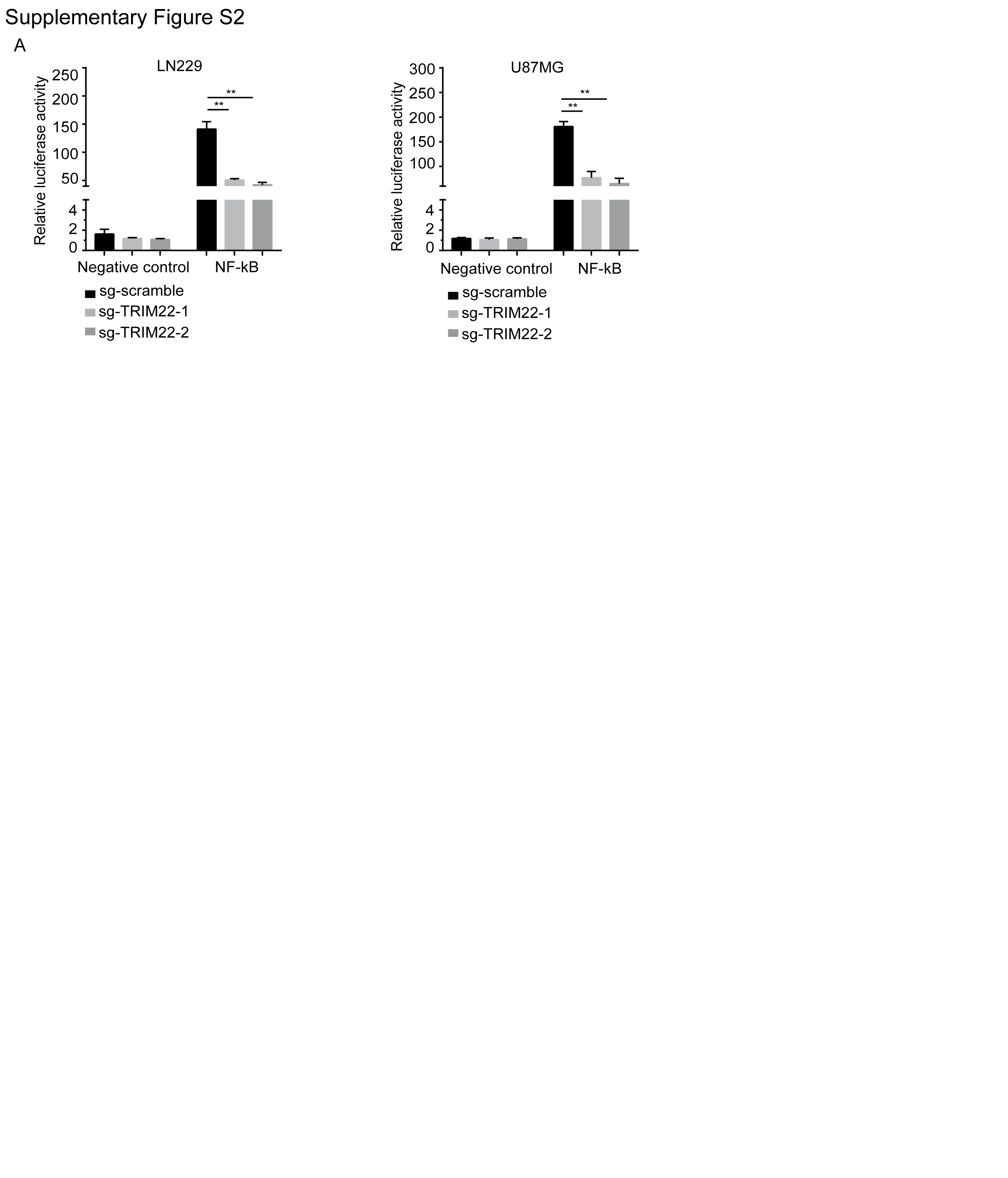

Supplement: Supplementary file 2 — Supplementary Figure S2 [file 41418_2020_606_MOESM2_ESM.tif]

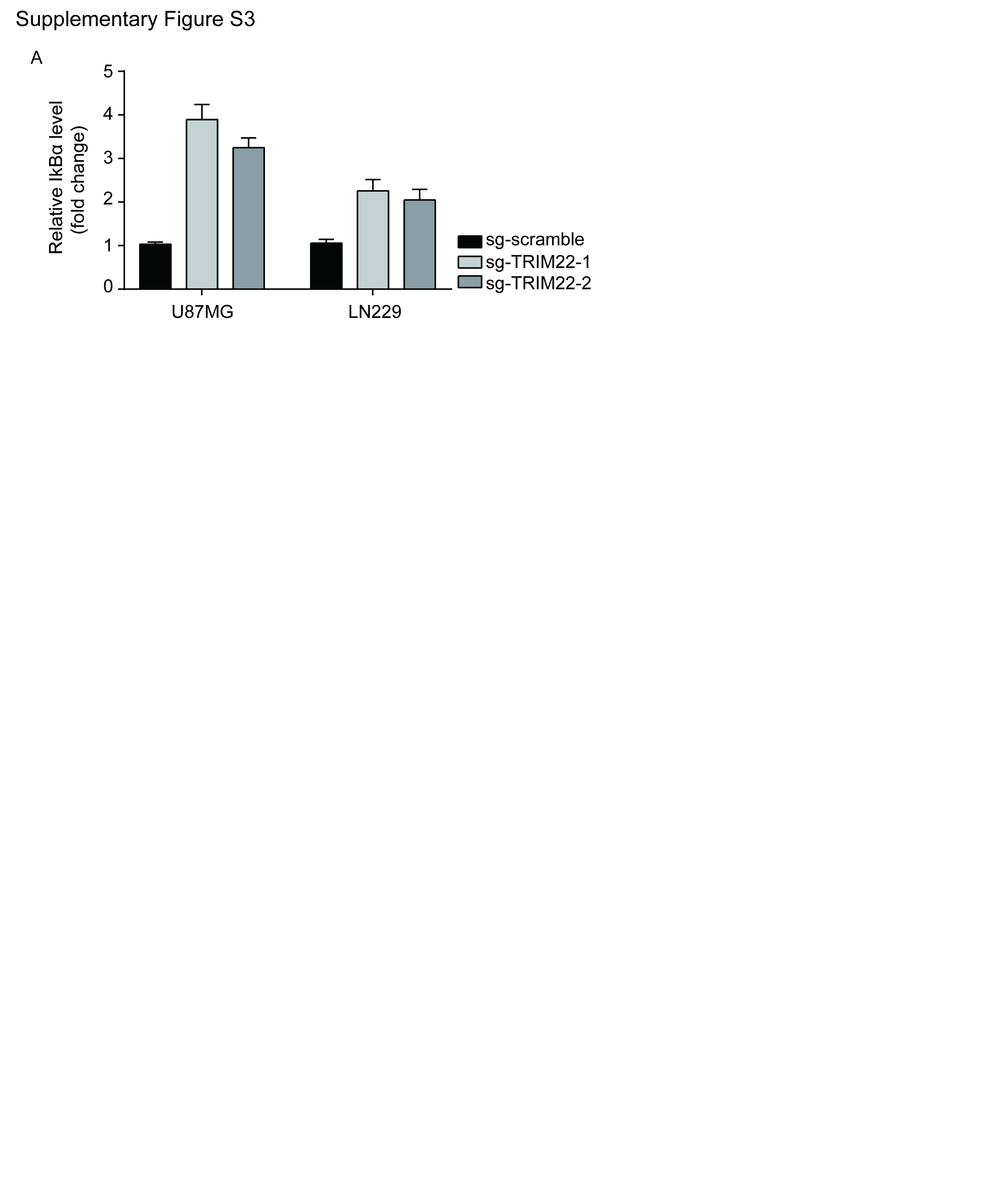

Supplement: Supplementary file 3 — Supplementary Figure S3 [file 41418_2020_606_MOESM3_ESM.tif]

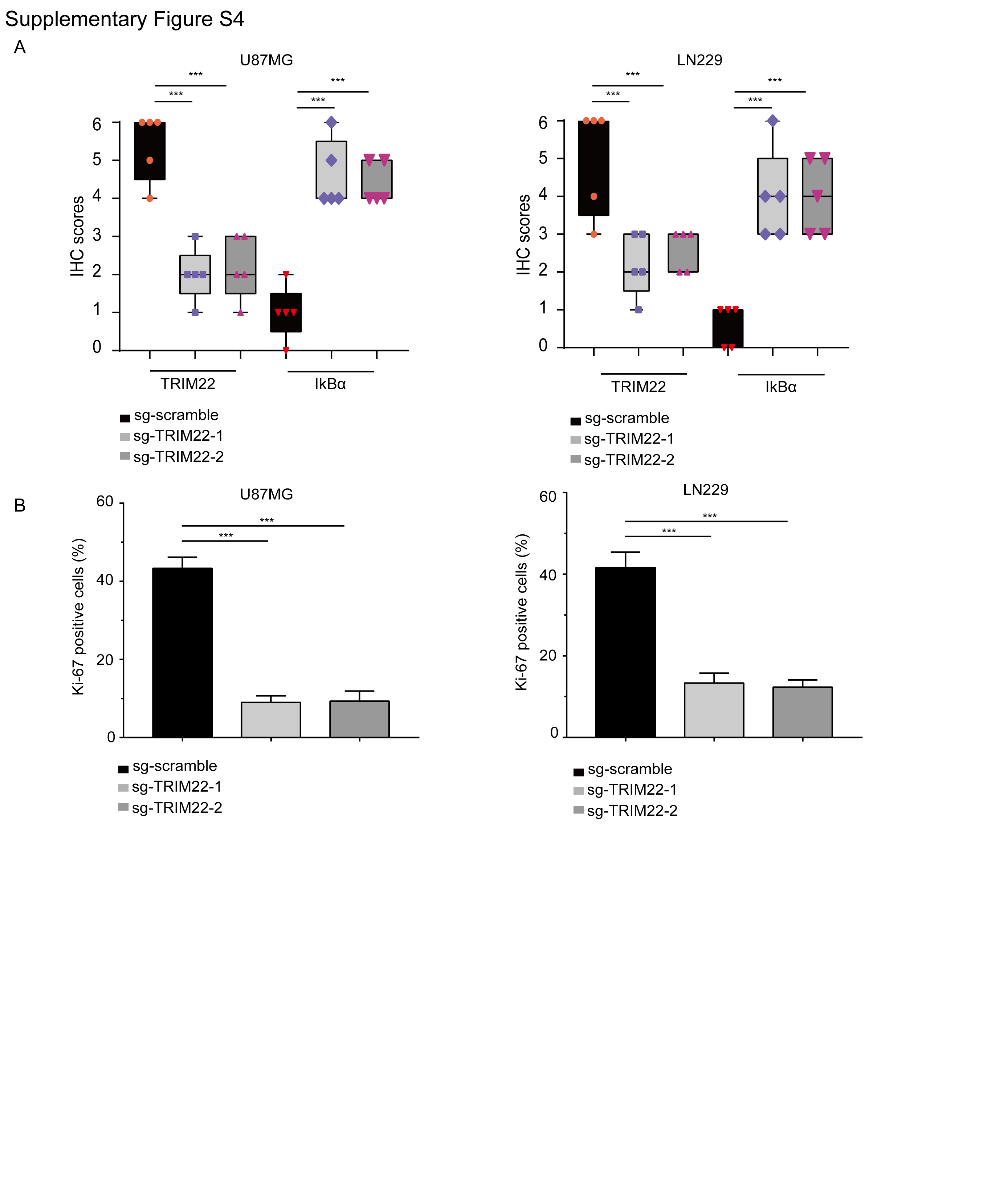

Supplement: Supplementary file 4 — Supplementary Figure S4 [file 41418_2020_606_MOESM4_ESM.tif]

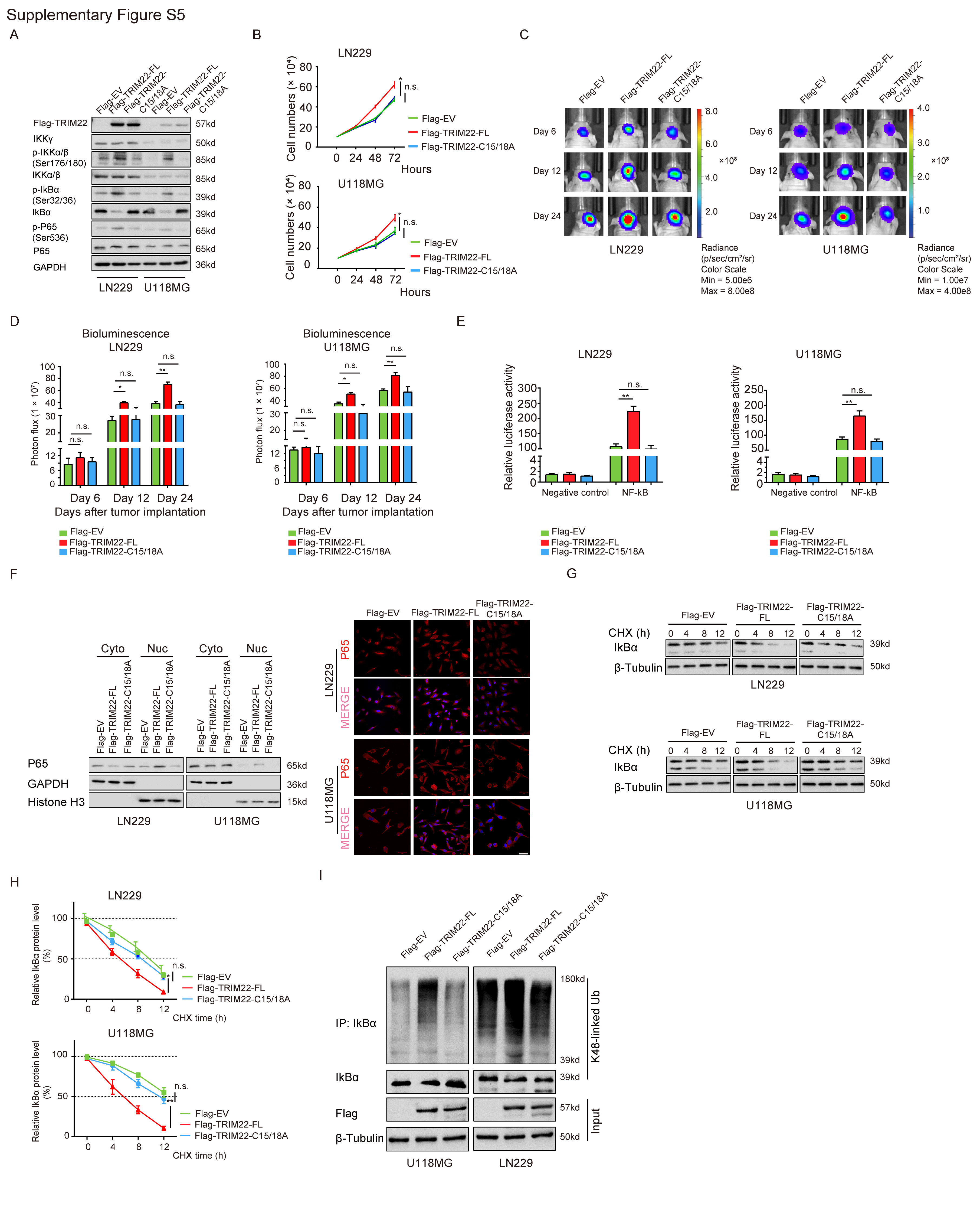

Supplement: Supplementary file 5 — Supplementary Figure S5 [file 41418_2020_606_MOESM5_ESM.tif]

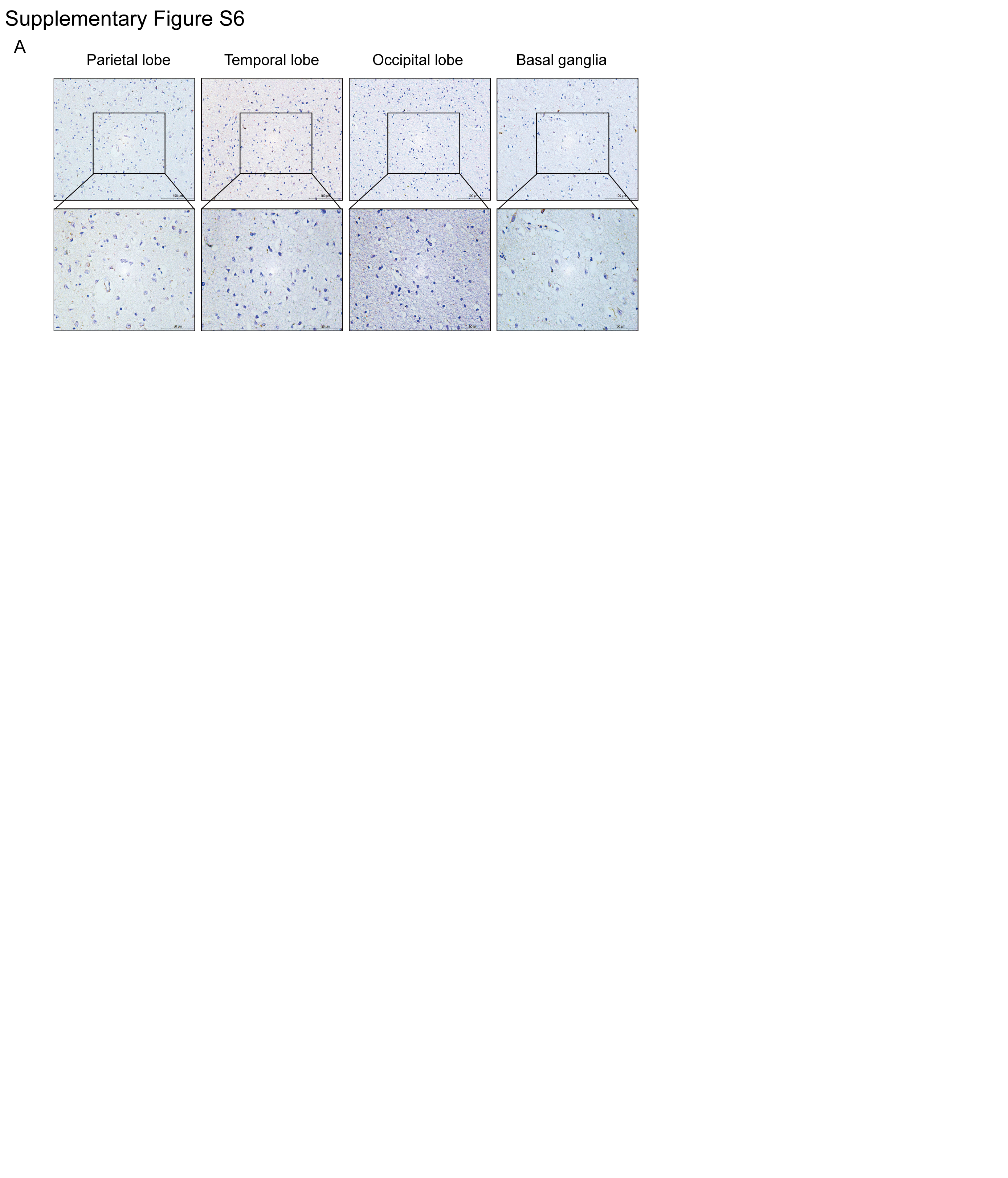

Supplement: Supplementary file 6 — Supplementary Figure S6 [file 41418_2020_606_MOESM6_ESM.tif]
