# Supplementary material for: TRIM22 activates NF-κB signaling in glioblastoma by accelerating the degradation of IκBα
Source: Cell Death Differ. 2020 Aug 19;28(1):367–81. doi: 10.1038/s41418-020-00606-w (PMC7853150; doi:10.1038/s41418-020-00606-w)
Supplement: Supplementary file 7 — Supplementary Table S1-S4 [file 41418_2020_606_MOESM7_ESM.docx]

**Supplementary Table S1. Oligonucleotide sets used in this study**

| **siRNAs or sgRNAs** | **Sequences** |
| --- | --- |
| si-NC | 5'-UUCUCCGAACGUGUCACGUTT-3' |
| si-*TRIM5* | 5'-GCTTCTGGAATCCTGGTTA-3’ |
| si-*TRIM21* | 5'-GCTCCCTCATCTACTCCTT-3’ |
| si-*TRIM22* | 5'-GCATAAACGAGGTGGTCAA-3' |
| si-*TRIM38* | 5'-GCTACTGCCACTTGTGTAT-3’ |
| si-*TRIM56* | 5'-CCACGTGGAGGTGTACAAT-3’ |
| sg-scramble | 5′-GCACTACCAGAGCTAACTCA-3′ |
| sg-*TRIM22*-1 | 5′-CCAGATGCCGATTAGGTCGG-3′ |
| sg-*TRIM22*-2 | 5′-CCGCATAAACGAGGTGGTCA-3′ |
| Puro-P2A-3Flag-hCas9 | Designed and constructed by OBiO Technology |

**Supplementary Table S2. Plasmids used in this study**

| **Plasmids** | **Resources** |
| --- | --- |
| pGL4.15-Control Vector | Promega |
| pGL4-SV40 Driven Renilla Luciferase Vector | Promega |
| pGL4.32[luc2P/NF-κB-RE/Hygro] Vector | Promega |
| pcDNA3.1-3xFlag-empty vector | OBiO Technology |
| pcDNA3.1-3xFlag-TRIM22-full length | OBiO Technology |
| pcDNA3.1-3xFlag-TRIM22-C15/18A | OBiO Technology |
| pcDNA3.1-3xFlag-TRIM22-1 | OBiO Technology |
| pcDNA3.1-3xFlag-TRIM22-2 | OBiO Technology |
| pcDNA3.1-3xFlag-TRIM22-3  pcDNA3.1-3xFlag-TRIM22-4 | OBiO Technology  OBiO Technology |
| pcDNA3.1-HA-empty vector | OBiO Technology |
| pcDNA3.1-HA-IkBα-full length | OBiO Technology |
| pcDNA3.1-HA-IkBα-1 | OBiO Technology |
| pcDNA3.1-HA-IkBα-2 | OBiO Technology |
| pcDNA3.1-HA-IkBα-3 | OBiO Technology |

**Supplementary Table S3. Primer sets used in this study**

| **Primer set** | **Primers** | **Sequence (5’-3’)** | **Product size (bp)** |
| --- | --- | --- | --- |
| ***TRIM22*** | F  R | 5'- GAGGTCAAGATGAGCCCACAG -3'  5'- GCTTTTCCTGACATTCCTTGACC -3' | **184** |
| ***IkBα***  ***GAPDH*** | F  R  F  R | 5'- AAGTGATCCGCCAGGTGAAG -3'  5'- CTGCTCACAGGCAAGGTGTA -3'  5’-GCACCGTCAAGGCTGAGAAC-3’  5’-TGGTGAAGACGCCAGTGGA-3’ | **188**  **138** |

**Supplementary Table S4. Association between TRIM22 expression and clinicopathological factors in glioma**

| Variables | No. of  cases | TRIM22 expression  low high | | *P* value |
| --- | --- | --- | --- | --- |
| **Age (year)** |  |  |  |  |
| <60 | 91 | 34 | 57 | 0.1311 |
| ≥60 | 21 | 4 | 17 |  |
| **Gender** |  |  |  |  |
| Male | 63 | 19 | 44 | 0.4217 |
| Female | 49 | 19 | 30 |  |
| **IDH1 Status** |  |  |  |  |
| WT | 51 | 8 | 43 | <0.001^***^  0.0054^**^  <0.001^***^ |
| MT | 61 | 30 | 31 |  |
| **ATRX Status**  Positive  Negative  **WHO Grade**  II  III  IV  Low grades  High grades | 76  36  30  31  51  30  82 | 19  19  20  11  7  20  18 | 57  17  10  20  44  10  64 |  |

WT: wild type; MT: mutant; LGG: low grade glioma, WHO II; HGG: high grade glioma, WHO III-IV. Fisher’s exact test or Chi-square test: *= *P* < 0.05; ***= *P* < 0.001.
