# Supplementary material for: TRIM22 activates NF-κB signaling in glioblastoma by accelerating the degradation of IκBα
Source: Cell Death Differ. 2020 Aug 19;28(1):367–81. doi: 10.1038/s41418-020-00606-w (PMC7853150; doi:10.1038/s41418-020-00606-w)
Supplement: Supplementary file 8 — Supplementary Materials and Methods, Figure Legends [file 41418_2020_606_MOESM8_ESM.docx]

**TRIM22 activates NF-κB signaling in glioblastoma by accelerating the degradation of IκBα**

**Supplementary Materials and Methods**

**Histologic evaluation system for IHC staining**

TRIM22 IHC was scored based on the intensity of the staining as follows: 0 (negative), 1 (weakly positive, light yellow), 2 (moderately positive, yellowish brown), and 3 (strongly positive, brown). The percentages of positive staining cells were also assessed and scored as follows: 0 (0%), 1 (1% to 33%), 2 (34% to 66%), and 3 (67% to 100%). The sum of the intensity and percentage scores was used as the final staining score. Low expression in tumors was defined as a total score of 0–3, and high expression was defined as a total score of 4–6.

**Antibodies used**

For western blotting, the following antibodies were used: TRIM22 (NBP1-81795, Novus Biologicals; Littleton, CO; 1:1000), IKKγ (ab178872, Abcam; Cambridge, MA; 1:1000), TRIM22 (ab68071, Abcam; 1:1000), IKKα/β (ab178870, Abcam; 1:1000), Histone-H3 (ab176842, Abcam; 1:1000), GAPDH (ab181603, Abcam; 1:10000), p-IKKα/β (Ser176/180) (#2697, Cell Signaling Technology; Beverly, MA; 1:1000), p-IκBα (Ser32/36) (#9246, Cell Signaling Technology; 1:1000), IκBα (#4814, Cell Signaling Technology; 1:1000), IκBα (#4812, Cell Signaling Technology; 1:1000), p-P65 (Ser536) (#3033, Cell Signaling Technology; 1:1000), P65 (#8242, Cell Signaling Technology; 1:1000), K48-linked Ub (#8081, Cell Signaling Technology; 1:1000), K63-linked Ub (#5621, Cell Signaling Technology; 1:1000), Flag-tag (#14793, Cell Signaling Technology; 1:1000), HA-tag (#3724, Cell Signaling Technology; 1:1000), β-Tubulin (#15115, Cell Signaling Technology; 1:1000), Flag-tag (F1804, Sigma-Aldrich; St. Louis, MO; 1:1000).

Ki-67 (ab92742, Abcam; 1:800) and P65(#8242, Cell Signaling Technology; 1:200) antibodies were used in IF assays.

TRIM22 (NBP1-81795, Novus Biologicals, Centennial, CO, USA; 1:200) and IκBα (#4814, Cell Signaling Technology; 1:200) antibodies were used in IHC.

Co-IPs were performed with non-specific IgG or antibodies specific for TRIM22 (ab68071, Abcam; 4 µL), IκBα (#4812, Cell Signaling Technology; 4 µL), Flag-tag (F1804, Sigma-Aldrich, St. Louis, MO, USA; 4 µL) and HA-tag (#3724, Cell Signaling Technology; 4 µL).

**Supplementary Figure Legends**

**Supplementary Fig. S1**

(a) Data mining in Oncomine to determine *TRIM5*, *TRIM21*, *TRIM22* and *TRIM38* mRNA levels in TCGA Brain datasets, and for *TRIM56* mRNA levels in Sun Brain datasets in GBM relative to non-neoplastic brain tissue samples.

**Supplementary Fig. S2**

(a) Luciferase activity for NF-κB luciferase or control reporter constructs in modified LN229 and U87MG cells. Student’s *t*-test: ***P* < 0.01

**Supplementary Fig. S3**

(a) Quantification of IκBα bands in Fig. 1B relative to GAPDH and to the control sg-scramble based on experiments performed in 3 independent biological replicates using ImageJ.

**Supplementary Fig. S4**

(a) Graphic representation of IHC scores for TRIM22 and IκBα levels in xenograft sections from control and sg-*TRIM22*-1 and -2 groups. (b) Graphic representation of IHC scores for Ki67 levels in xenograft sections from indicated groups. Student’s *t*-test: ****P* < 0.001

**Supplementary Fig. S5**

(a) Western blot analysis to evaluate components of the NF-κB pathway in lysates prepared from LN229- and U118MG-EV, TRIM22-FL and TRIM22-C15/18A cell populations. GAPDH was used as the loading control. (b) Growth curves generated using cell counting for transfected cells in (a). (c, d) Representative images and quantification of *in vivo* luciferase bioluminescence from indicated cells orthotopically implanted into the brains of nude mice at days 6, 12, and 24 after injection. (e) Luciferase activity for NF-κB luciferase or control reporter constructs in modified LN229 and U118MG cells. (f) Western blot analysis of cytoplasmic (Cyto) and nuclear (Nuc) fractions prepared from indicated cells. Immunofluorescence for P65 in modified LN229 and U118MG cells showing cellular localization. Scale bars, 20 μm. (g) Western blot to detect IκBα levels after 0 h, 4 h, 8 h, and 12 h of cycloheximide (CHX; 25 μg/mL) treatment in modified LN229 and U118MG cells compared with controls. (h) Line graph showing IκBα protein levels normalized to β-tubulin and to 0 h at the indicated time points. (i) *In vivo* ubiquitination assay of IκBα. Student’s *t*-test: n.s. = not significant, **P* < 0.05, ***P* < 0.01.

**Supplementary Fig S6.**

(a) IHC performed on non-neoplastic brain tissues derived from four regions of the brain, including parietal, temporal, and occipital lobes, and the basal ganglia. Scale bar = 100 µm (top) or 50 µm (bottom).

**Supplementary Table S1.** Oligonucleotide sets used in this study.

**Supplementary Table S2.** Plasmids used in this study.

**Supplementary Table S3.** Primer sets used in this study.

**Supplementary Table S4.** Association between TRIM22 expression and clinicopathological factors in glioma.
